# Editorial: Digital Skills and Life-Long Learning: Digital Learning as a New Insight of Enhanced Learning by the Innovative Approach Joining Technology and Cognition

**DOI:** 10.3389/fpsyg.2018.02621

**Published:** 2018-12-19

**Authors:** Dina Di Giacomo, Pierpaolo Vittorini, Pilar Lacasa

**Affiliations:** ^1^Department of Life, Health and Environment Sciences, University of L'Aquila, L'Aquila, Italy; ^2^Faculty of Humanities, University of Alcalá, Alcalá de Henares, Madrid, Spain

**Keywords:** digital, lifelong learning, digital skill, technology, ICT

Digital skills represent enhanced learning abilities within which cognition and technology interact to model the knowledge processes in aging populations. Since they are digital natives, new generations are tech savvy, using improved learning capabilities. Adults and older generations do not possess native technological competence. However, they are learning digital skills to improve their lives and to age well in a digital world.

Digital and technological innovations are impacting the daily living of people influencing significantly the demographic changes. New kind of jobs, new way to manage the healthcare, education, and social contexts. In the “White Paper on the Future of Europe,” the European Commission (2017) highlights that it “is likely that most children entering primary school today will end up working in new job types that do not yet exist” and that coping with this “will require a massive investment in skills and a major rethink of education and lifelong learning system.”

The Research Topic aimed to elaborate the benefits of digital skills such as to overcoming the barriers of technology, investigating the human factor in digital life-long learning, advancing the cognitive technology debate, overcoming the dichotomies between technology and psychology, and emphasizing the improvements to human knowledge and wellness. The studies' focus was on investigating technology's impact on cognitive and intellectual processes by highlighting the intensity with which technology can change and/or enhance cognitive/behavioral functioning over a lifetime. Multidisciplinary research has been the favored scientific approach.

The Research Topic Issue is composed of original research, as well as a technical report and review. The original research addressed relevant digital inclusion themes and self-efficacy in ICT and teaching practices. The cognitive training game focused on the adult population to see the impact of digital technology on aging adults' cognition and behaviors.

Several researchers have provided evidence of the potential for technology to improve cognitive development and learning outcomes in children. Many studies have highlighted the efficacy of technology in an educational context to promote innovative learning and greater cognitive autonomy (Zimmerman and Tsikalas, 2005; Alvermann et al., 2006; Di Giacomo et al., 2016a,b). Using an evidence-based approach, Di Giacomo et al.'s study investigated digital learning as smart learning in children by examining the influence of digital experiences on children's cognitive development. Chao and Yu's study investigated Internet access and cyberbullying behavior in adolescents, and Rodriguez-Andres et al. focused on how videogames influence children's navigation skills. All of the research listed showed the impact of technology on daily living of people highlighting the changing in lifelong learning.

Older adults' abilities to use digital solutions and tools is a crucial issue, because low adherence to digital living creates a barrier in daily living, which reduces quality of life, independence, autonomy, and mental health. Moreover, such tools could effectively enhance medical care for the elderly. However, the fear of technology is more prevalent among older generations who did not grow up with computers, complicated acronyms, or digital games.

While the benefits of learning about computers and applications are abundant, on the negative side, learning can be stressful for people due to cognitive as well as psychological factors. This affect is commonly identified as “computer anxiety” (Cambre and Cook, 1985; Desai and Richards, 1998; Thatcher and Perrewe, 2002). Martínez-Alcalá et al.'s study aimed at the digital inclusion of older adults, whereas Hou et al. dealt with technophobia as barrier to digital living in adulthood, and Lu et al.'s study developed a cognitive training game for older people. The last original research from Hatlevik and Hatlevik's was dedicated to the impact of technology on adults and their teaching practices.

Gibson's technology report designed a new framework to unobtrusively observe and analyse knowledge and skills-in-action by continuously collecting data from individuals as they interacted with digital assets either alone or on problem-solving teams.

Finally, the review by Rossignoli-Palomeque et al. debated the scientific concept of brain training in children and youth, specifically its efficacy in populations with neurological diseases.

## Author Contributions

DD, PV and PL wrote the Editorial.

### Conflict of Interest Statement

The authors declare that the research was conducted in the absence of any commercial or financial relationships that could be construed as a potential conflict of interest.
